# Current Clinical Practices and Digital Technology Adoption in Provisional Prosthodontics: A Cross-Sectional Survey Among Romanian Dental Practitioners

**DOI:** 10.3390/dj14080489

**Published:** 2026-08-06

**Authors:** George Ion, Ştefan Milicescu, Ștefan-Dimitrie Albu, Dan Alexandru Slăvescu, Cristina-Crenguţa Albu, Claudia Florina Bogdan-Andreescu

**Affiliations:** 1 Department of Prosthetic Dentistry, Faculty of Dentistry, “Carol Davila” University of Medicine and Pharmacy, 020021 Bucharest, Romania; george_ion@umfcd.ro; 2 Department of Dental Esthetics, Faculty of Dentistry, “Carol Davila” University of Medicine and Pharmacy, 020021 Bucharest, Romania; stefan.milicescu@umfcd.ro; 3 Department of Periodontology, Faculty of Dentistry, “Carol Davila” University of Medicine and Pharmacy, 020021 Bucharest, Romania; 4 Department of Dentistry, Faculty of Medicine and Pharmacy, University of Oradea, 410073 Oradea, Romania; 5 Department of Genetics, Faculty of Dentistry, “Carol Davila” University of Medicine and Pharmacy, 020021 Bucharest, Romania; cristina.albu@umfcd.ro; 6 Department of Specialty Disciplines, “Titu Maiorescu” University, 031593 Bucharest, Romania; claudia.andreescu@prof.utm.ro

**Keywords:** provisional restorations, temporary crowns, CAD/CAM, digital dentistry, additive manufacturing, 3D printing, prosthodontics, survey study

## Abstract

**Background/Objectives:** Provisional restorations are essential for maintaining oral function, esthetics, periodontal health, and patient comfort during prosthodontic treatment. This study evaluated current clinical practices, material preferences, fabrication techniques, and digital technology adoption among Romanian dental practitioners. **Methods:** A cross-sectional survey was conducted between January and March 2025. The questionnaire was sent directly to a predefined closed list of 150 licensed dental practitioners, of whom 95 submitted complete responses (response rate: 63.3%). The questionnaire assessed professional characteristics, materials and fabrication techniques used for provisional restorations, attitudes toward digital technologies, educational sources, and clinical complications. Data were analyzed using descriptive statistics, association tests, and exploratory multivariable logistic regression. **Results:** Composite resin (77.9%) and acrylic resin (65.3%) were the most frequently reported materials. Direct fabrication was used by 51.6% of respondents, while 42.1% used indirect techniques. An affirmative response to the general item concerning current digital technology use was provided by 47.4% of participants. Faster and more efficient fabrication and superior intraoral adaptation were the principal perceived advantages of 3D-printed restorations, whereas high equipment and material costs represented the main perceived disadvantage. The exploratory multivariable analysis identified no statistically significant independent associations with digital technology utilization. Debonding (44.2%) and fracture (42.1%) were the most frequently reported complications. **Conclusions:** Among the surveyed practitioners, conventional materials and fabrication techniques remained widely used, while digital workflows showed incomplete integration. Larger, representative studies using clearly defined technology-specific outcomes are needed to evaluate the determinants and clinical implications of digital adoption in provisional prosthodontics.

## 1. Introduction

Provisional restorations are an essential component of contemporary prosthodontic treatment, serving not only as temporary substitutes for definitive restorations but also as important therapeutic and diagnostic tools. Their clinical functions include protection of prepared teeth, preservation of periodontal health, maintenance of occlusal stability, optimization of esthetics and phonetics, and evaluation of functional parameters before definitive rehabilitation, applicable to both fixed and removable provisional solutions [1,2,3,4]. In implant prosthodontics, provisional restorations additionally contribute to peri-implant soft tissue conditioning and the development of favorable emergence profiles, directly influencing long-term esthetic and biological outcomes [5,6,7].

The clinical performance of provisional restorations is greatly influenced by the materials and fabrication methods employed. Conventional materials, including polymethyl methacrylate (PMMA), polyethyl methacrylate (PEMA), and bis-acryl composite resins, remain widely used because of their accessibility, acceptable mechanical properties, and ease of clinical manipulation [8,9,10]. However, limitations such as polymerization shrinkage, marginal deterioration, fracture susceptibility, color instability, and reduced long-term durability continue to challenge clinicians, particularly in cases requiring extended provisionalization periods [11,12,13,14].

The rapid digitalization of dentistry has substantially transformed restorative workflows over the past two decades. Computer-aided design and computer-aided manufacturing (CAD/CAM) technologies have enabled highly standardized fabrication processes characterized by improved accuracy, reproducibility, and efficiency [15]. CAD/CAM-milled provisional restorations have demonstrated favorable marginal adaptation, enhanced mechanical properties, and superior material homogeneity compared with conventionally fabricated alternatives [16]. Simultaneously, additive manufacturing technologies have appeared as promising alternatives, offering reduced material waste, shorter production times, and the ability to fabricate geometrically complex restorations through layer-by-layer construction [17].

Despite the growing body of laboratory evidence supporting digital fabrication techniques, the translation of these technologies into routine clinical practice remains uneven. Existing studies have predominantly focused on material properties, manufacturing accuracy, and in vitro performance, whereas considerably less attention has been devoted to understanding how dental practitioners integrate digital workflows into everyday prosthodontic practice [18,19]. Factors such as financial investment, access to equipment, collaboration with dental laboratories, professional training, and perceived clinical benefits may substantially influence technology adoption [20,21,22].

Furthermore, evidence regarding the implementation of digital technologies in provisional prosthodontics within Eastern European countries remains limited [23,24]. Romania represents a particularly relevant setting because of the rapid expansion of digital dentistry infrastructure alongside the continued reliance on conventional restorative approaches [25]. Understanding practitioners’ preferences, clinical decision-making processes, and perceived barriers to digital adoption may provide valuable information for educational institutions, professional organizations, dental laboratories, and technology developers [20,26,27].

Recent Romanian studies have begun to explore digital technology adoption in dentistry from complementary perspectives. Burde et al. assessed the use of CAD/CAM technology among Romanian dental technicians, reporting that 54.8% currently employed some form of CAD/CAM in their laboratories, although high initial investment remained the principal barrier to wider implementation [28]. More recently, Cozmescu et al. investigated Romanian dentists’ perspectives on the integration of artificial intelligence into clinical practice, finding that 80.5% of respondents already used digital techniques in daily practice and that the majority expressed willingness to adopt AI-based solutions, while financial and data security concerns were identified as major obstacles [29]. Similarly, Andronic et al. examined the adoption of teledentistry among Romanian practitioners in Sibiu County, identifying implementation barriers through association rule mining [30]. However, none of these studies addressed material selection, fabrication techniques, or the specific adoption of CAD/CAM and additive manufacturing technologies in the context of provisional restorations, underscoring the absence of a survey specifically focused on this area of prosthodontic practice in Romania.

To date, limited evidence is available regarding the adoption of digital technologies in provisional prosthodontics among Romanian dental practitioners. Furthermore, no previous survey conducted among Romanian dental practitioners has comprehensively evaluated material selection, fabrication techniques, CAD/CAM and 3D-printing utilization, educational influences, and clinical complications within a single analytical framework. Therefore, the present study aimed to investigate current clinical practices related to provisional restorations among Romanian dental practitioners, with particular emphasis on material selection, fabrication techniques, utilization of CAD/CAM and three-dimensional printing technologies, continuing professional education, and commonly encountered clinical complications. In addition, the study sought to identify factors associated with the adoption of digital workflows in provisional prosthodontics. The working hypothesis was that digital technology use would be reported by a substantial proportion of respondents and would be associated with selected practitioner- and practice-related characteristics.

## 2. Materials and Methods

### 2.1. Study Design

This cross-sectional observational study was conducted to investigate current clinical practices related to provisional restorations among Romanian dental practitioners. The survey explored material selection, fabrication techniques, utilization of digital technologies, continuing professional education, and complications associated with provisional prosthodontic treatment.

Data were collected between January and March 2025 using a structured online questionnaire distributed directly to a closed list of 150 licensed dental practitioners identified through the professional networks of the research team. The study followed the recommendations of the Strengthening the Reporting of Observational Studies in Epidemiology (STROBE) guidelines for cross-sectional studies (File S1 and Table S1).

### 2.2. Study Population and Sampling

The target population comprised licensed dentists actively engaged in clinical practice in Romania. Participants were recruited using non-probability convenience sampling. A closed distribution list of 150 potentially eligible dental practitioners was compiled through the professional networks of the research team. The survey invitation and questionnaire link were sent directly and individually to these 150 practitioners and were not posted on unrestricted public social media platforms or open-access groups.

The inclusion criteria were: (1) holding a valid qualification to practice dentistry in Romania; (2) being actively engaged in clinical dental practice during the study period; (3) voluntarily agreeing to participate, as indicated by submission of the completed questionnaire; and (4) submitting responses to all mandatory questionnaire items.

The exclusion criteria were: (1) dental students who were not yet licensed practitioners; (2) dental technicians and other oral healthcare professionals who were not licensed dentists; (3) dentists who were not actively engaged in clinical practice during the study period; (4) declining to participate; (5) submitting an incomplete questionnaire; and (6) participation in the pilot-testing phase. Responses obtained during pilot testing were not included in the final dataset.

Of the 150 dental practitioners invited to participate, 95 submitted fully completed questionnaires and were included in the final analysis, yielding a response rate of 63.3%. Participants represented different professional categories, levels of clinical experience, practice settings, and urban and rural environments. No incentives or financial compensation were offered.

No formal a priori sample-size calculation was performed because the study was designed as an exploratory survey using non-probability convenience sampling. The final sample size was determined by the number of fully completed questionnaires returned by practitioners from the predefined closed distribution list during the study period. Given the non-probability sampling strategy and the relatively modest achieved sample, the findings should be interpreted as preliminary and exploratory and should not be considered nationally representative or suitable for definitive subgroup comparisons.

### 2.3. Ethical Considerations

Participation was entirely voluntary and anonymous. Before accessing the questionnaire, respondents received information regarding the study objectives, the anonymous nature of data collection, and the intended scientific use of the results. Submission of the completed questionnaire was considered to indicate informed consent.

The study consisted exclusively of a non-interventional online survey of licensed dental practitioners and did not involve patients, clinical interventions, biological specimens, sensitive health information, or the collection of directly identifiable personal data. No personally identifiable information was collected, stored, or processed. The study was conducted in accordance with the ethical principles of the Declaration of Helsinki and applicable data-protection requirements, including Regulation (EU) 2016/679 and Romanian Law No. 190/2018 [31]. Based on the anonymous, voluntary, and non-interventional nature of the survey, the authors determined that formal ethical review was not required.

### 2.4. Questionnaire Development and Validation

The questionnaire was developed by the research team following an extensive review of the contemporary literature on provisional restorations, CAD/CAM technologies, additive manufacturing, and digital workflows in prosthodontics. The instrument was designed to evaluate current clinical practices, material preferences, fabrication techniques, digital technology adoption, perceived barriers, and educational needs related to provisional restorative treatment.

To establish content validity and face validity, the preliminary questionnaire was reviewed by three academic experts in prosthodontics and digital dentistry. The experts evaluated the relevance, clarity, comprehensiveness, and appropriateness of each item in relation to the study objectives. Their recommendations resulted in minor modifications to item wording, sequencing, and overall questionnaire structure.

Subsequently, the questionnaire underwent pilot testing among a small group of practicing dentists who were not included in the final study population. The pilot phase assessed comprehensibility, feasibility, completion time, and the clarity of instructions and response options. Feedback obtained during pilot testing was incorporated into the final version of the instrument. Responses collected during the pilot phase were excluded from the final dataset.

The first section of the questionnaire collected demographic and professional information, including sex, practice environment (urban or rural), professional status (general dental practitioner, specialist, or resident), years of clinical experience, and type of practice (private or insurance-contracted practice).

The second section explored clinical approaches to provisional restorations and the adoption of digital technologies in prosthodontics. Specifically, participants were asked about the materials used for provisional restorations, fabrication techniques, factors influencing material selection, interest in emerging restorative materials and technologies, perceived advantages and disadvantages of additive manufacturing technologies, perceived benefits and limitations of additive manufacturing technologies, and the impact of digital workflows on restoration quality. Additional items investigated the current utilization of digital technologies in daily clinical practice, perceived patient acceptance of digitally fabricated provisional restorations, sources of continuing professional education, strategies employed to improve clinical outcomes, and complications frequently encountered during provisional prosthodontic treatment.

The questionnaire included a general item asking whether respondents currently used digital technologies for the fabrication of provisional restorations. No additional operational definition or separate checklist of qualifying technologies was provided for this item. Consequently, the response reflected each participant’s individual interpretation of a digital provisional-restoration workflow.

The questionnaire consisted predominantly of closed-ended multiple-choice questions with single-response options. One item addressing the materials used for provisional restorations permitted multiple responses to reflect the diversity of materials routinely employed in clinical practice. Completion of the questionnaire required approximately 5–10 min. Participation was voluntary and anonymous, and respondents were free to discontinue the survey at any time prior to submission.

Several measures were taken to reduce potential bias. The questionnaire was developed following a review of the relevant literature and was evaluated by three academic experts in prosthodontics and digital dentistry for content and face validity. It was subsequently pilot-tested among practicing dentists to assess clarity, comprehensibility, feasibility, and completion time. The dentists involved in pilot testing were not included in the final study sample. All invited practitioners received the same questionnaire, instructions, response options, and data-collection timeframe. Participation was voluntary and anonymous, and no incentives were provided.

A total of 150 practitioners received the questionnaire invitation through the predefined closed distribution list. Survey responses were monitored throughout the data-collection period using the administrative interface of the online survey platform. The number of submitted questionnaires and their completion status were reviewed periodically. Of the 150 invited practitioners, 95 submitted fully completed questionnaires and were retained for statistical analysis, corresponding to a response rate of 63.3%. Incomplete submissions and responses obtained during pilot testing were excluded. Because the survey was anonymous, no personally identifiable information, IP addresses, or other direct identifiers were used to monitor participation.

### 2.5. Statistical Analysis

Data were analyzed using IBM SPSS Statistics software, version 29 (IBM Corp., Armonk, NY, USA). Descriptive statistics were used to summarize participants’ demographic characteristics and questionnaire responses. Categorical variables were presented as absolute frequencies and percentages.

Associations between categorical variables were evaluated using Pearson’s chi-square test. Effect sizes were estimated using Cramer’s V coefficient. Binary logistic regression was performed as an exploratory analysis to examine potential associations between practitioner- and practice-related characteristics and the reported use of digital technologies for provisional restorations. Candidate predictors were selected based on their clinical relevance, the existing literature, and the study objectives. Results were reported as odds ratios (ORs) with corresponding 95% confidence intervals (95% CIs). Statistical significance was established at a two-sided *p*-value < 0.05.

Before regression analysis, multicollinearity among the candidate predictors was assessed using variance inflation factors (VIFs), and no evidence of problematic multicollinearity was identified. The apparent discrimination of the fitted model was summarized using the area under the receiver operating characteristic curve (AUC), while Nagelkerke’s pseudo-R^2^ was reported as a descriptive measure of model fit. No internal or external model validation was performed.

Because the model included 12 predictor parameters relative to only 45 outcome events, the events-per-parameter ratio was below commonly recommended thresholds. Accordingly, the regression analysis was considered exploratory and hypothesis-generating rather than confirmatory. Individual odds ratios, confidence intervals, AUC, and Nagelkerke’s R^2^ were interpreted cautiously and were not considered evidence of stable independent effects or reliable predictive performance.

## 3. Results

### 3.1. Demographic Characteristics of Participants

A total of 150 questionnaires were distributed electronically, and 95 complete responses were included in the final analysis, corresponding to a response rate of 63.3%.

**Figure 1:**
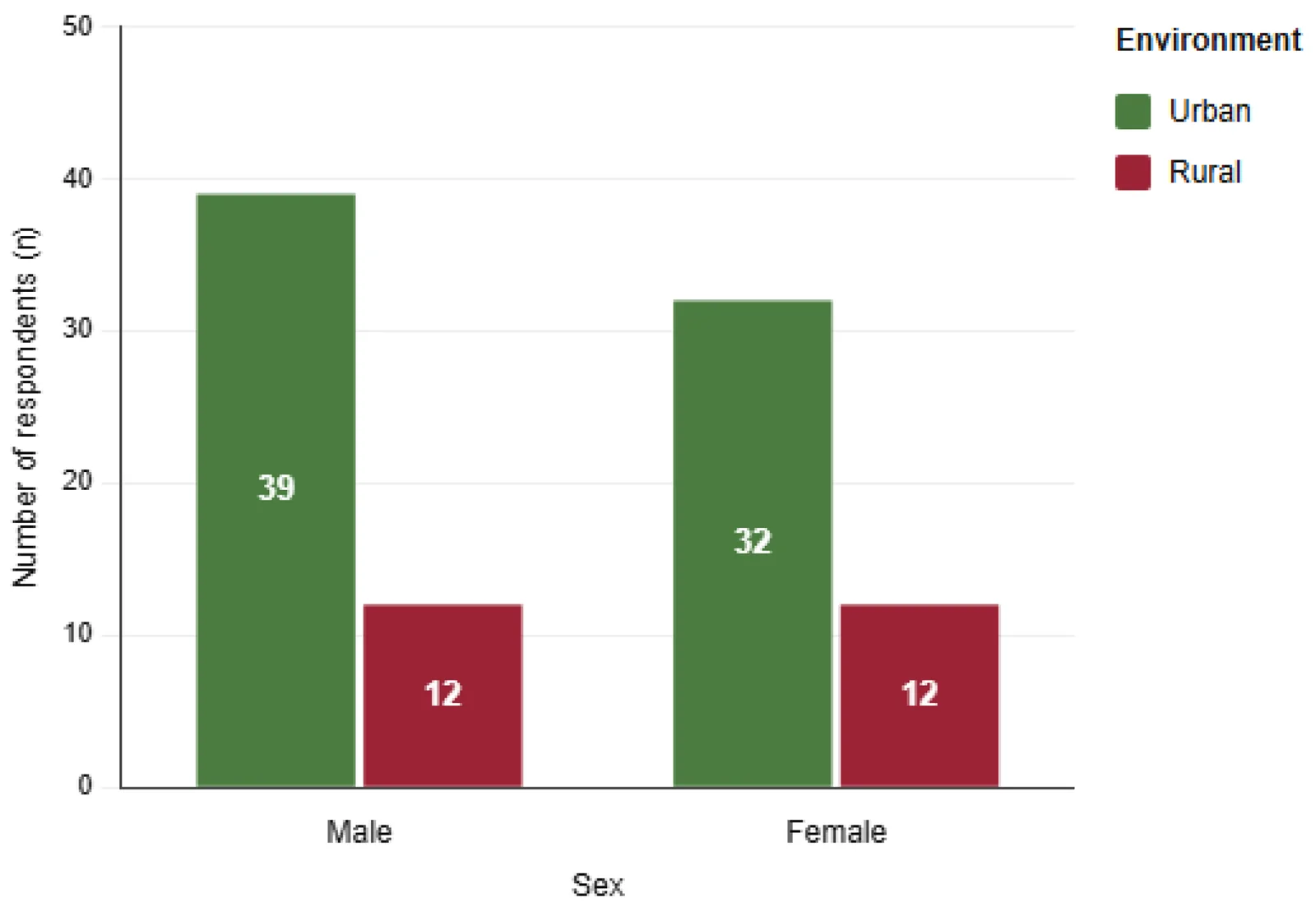
Distribution of respondents according to sex and practice environment (urban/rural).

The distribution of respondents according to sex and practice environment is illustrated in Figure 1. Most participants practiced in urban areas regardless of sex.

The relationship between practice environment and type of practice is presented in Table 1. Although private practice was more frequently reported among urban respondents, no statistically significant association was identified between practice environment and type of practice (χ^2^ = 0.12, *p* = 0.730, Cramer’s V = 0.035).

### 3.2. Professional Characteristics of Participants

**Figure 2:**
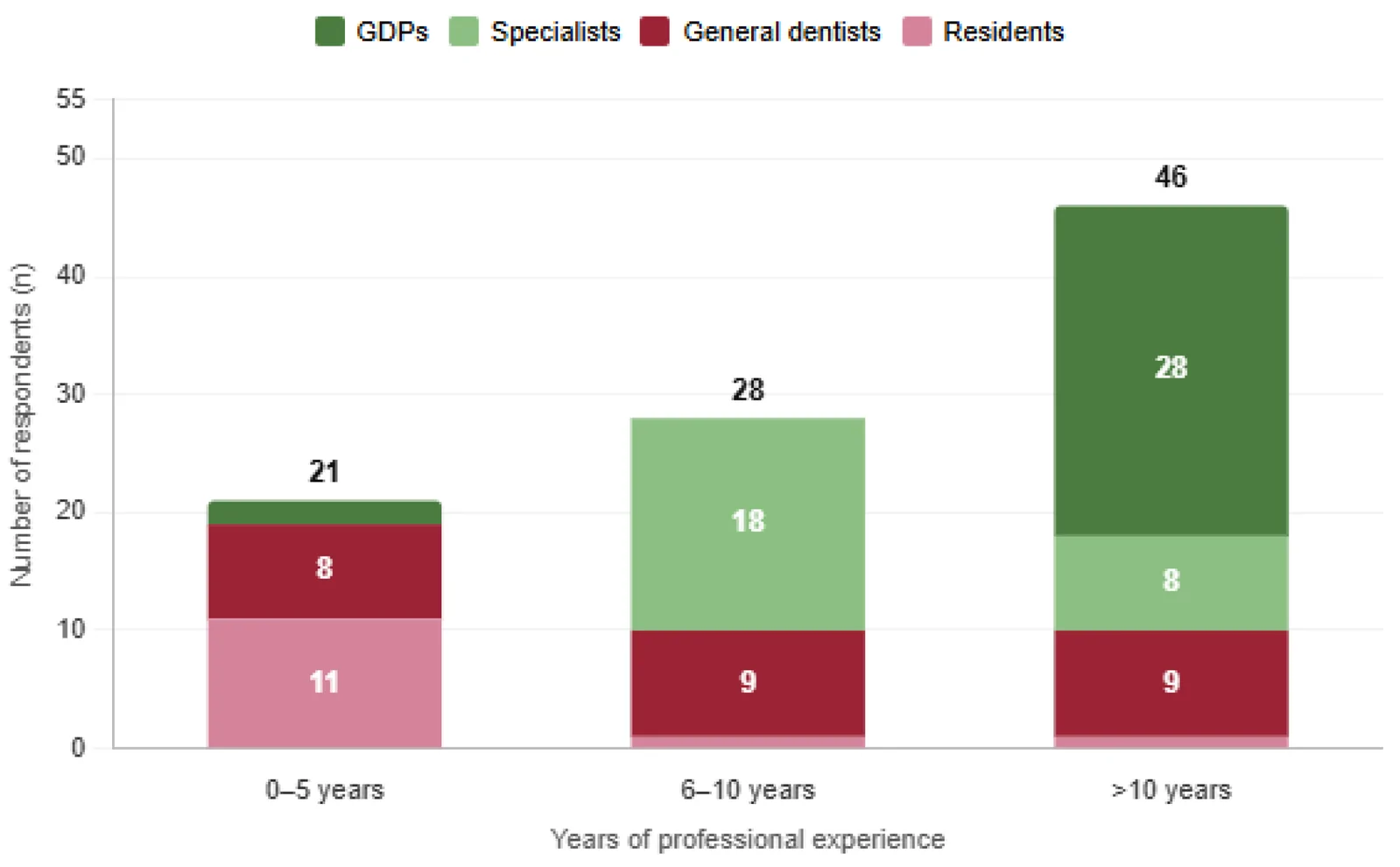
Distribution of respondents according to years of clinical experience and professional status. Note: General dental practitioners (GDPs) predominated among practitioners with more than 10 years of professional experience, whereas residents were concentrated within the early stages of professional activity. Specialists were represented across all experience categories, with the highest frequency observed among practitioners with 6–10 years of experience.

The distribution of respondents according to professional status and years of clinical experience is presented in Figure 2. General dental practitioners predominated among participants with more than 10 years of professional experience, whereas residents were concentrated within the early stages of professional activity. Specialists were represented across all experience categories.

Participants’ responses concerning materials, selection criteria, fabrication techniques, attitudes toward emerging technologies, perceived advantages and disadvantages of 3D printing, current digital technology use, patient acceptance, professional information sources, improvement strategies, and clinical complications are consolidated in Table 2.

### 3.3. Materials Used for Provisional Restorations

As shown in Table 2, composite resin and acrylic resin were the most frequently reported materials, whereas metallic provisional restorations were less commonly used.

Associations between respondents’ characteristics and material selection are summarized in Table 3. No statistically significant relationships were observed between practice environment or professional status and the use of the investigated materials. The association between professional status and the use of polycarbonate crowns did not reach the prespecified threshold for statistical significance (*p* = 0.051).

### 3.4. Criteria Influencing Material Selection

The principal criteria influencing material selection are presented in Table 2. Cost and resistance were the most frequently reported determinants of material choice.

### 3.5. Preferred Techniques for Provisional Restoration Fabrication

The direct technique represented the most commonly reported approach for provisional restoration fabrication, followed by the indirect technique (Table 2).

### 3.6. Attitudes Toward Emerging Technologies and Materials

Most respondents expressed either a positive attitude toward emerging technologies or a willingness to adopt them provided that sufficient clinical evidence becomes available (Table 2).

### 3.7. Perceptions of Additive Manufacturing Technologies

Faster and more efficient fabrication and superior adaptation within the oral cavity were identified as the principal perceived advantages of 3D-printed provisional restorations, whereas high equipment and material costs represented the most frequently reported disadvantage (Table 2).

### 3.8. Perceived Influence and Current Utilization of Digital Technologies

Most respondents considered that digital technologies positively influence the quality of provisional restorations. However, only 47.4% provided an affirmative response to the general questionnaire item concerning the current use of digital technologies for the fabrication of provisional restorations (Table 2).

### 3.9. Factors Associated with Digital Technology Utilization

The results of the exploratory multivariable logistic regression analysis are presented in Table 4. None of the investigated variables showed a statistically significant independent association with digital technology utilization at the prespecified α level of 0.05. The estimates were characterized by considerable uncertainty, as reflected by the wide confidence intervals.

The model yielded an apparent AUC of 0.714 and a Nagelkerke’s R^2^ of 0.182 in the analyzed sample. However, because the model included 12 predictor parameters relative to only 45 outcome events and underwent no internal or external validation, these measures should not be interpreted as evidence of reliable predictive or discriminatory performance. The analysis is presented solely for exploratory and hypothesis-generating purposes.

### 3.10. Perceived Patient Acceptance of Digitally Fabricated Provisional Restorations

Respondents’ perceptions regarding patient acceptance of digitally fabricated provisional restorations are presented in Table 2. Most participants considered that patient acceptance is influenced primarily by financial considerations. More than half of the respondents indicated that patients might be interested in digitally fabricated restorations but that additional costs could limit their accessibility. Only a small proportion believed that most patients would readily accept these restorations despite the increased treatment costs.

### 3.11. Sources of Professional Information

The primary sources of professional information used by respondents are summarized in Table 2. Scientific congresses and professional meetings represented the most frequently reported educational resources, followed by continuing education courses. Internet resources and scientific journals and textbooks were less frequently reported as the principal source of professional knowledge.

### 3.12. Measures Implemented to Optimize Provisional Prosthodontic Practice

The strategies implemented by respondents to improve provisional prosthodontic treatment are presented in Table 2. Additional professional training was the most commonly reported measure, followed by collaboration with specialized dental laboratories and investments in updated equipment and restorative materials.

### 3.13. Frequently Encountered Complications Associated with Provisional Restorations

The complications most frequently encountered in clinical practice are presented in Table 2. Debonding and fracture represented the predominant complications reported by respondents, whereas periodontal complications were less frequently reported.

## 4. Discussion

### 4.1. Principal Findings

The present study provides insight into current provisional prosthodontic practices among Romanian dental practitioners and highlights the ongoing transition toward digital restorative workflows. Several important findings emerged. First, composite and acrylic resins remain the predominant materials used for provisional restorations. Second, direct fabrication techniques continue to represent the preferred clinical approach. Third, although respondents generally expressed favorable attitudes toward digital technologies, 47.4% provided an affirmative response to the general questionnaire item concerning current digital technology use. Finally, respondents frequently identified economic considerations, professional education, and perceived technological benefits as factors relevant to the implementation of digital solutions.

These findings suggest that provisional prosthodontics currently exists in a transitional phase in which conventional methods remain dominant while favorable perceptions of digital technologies were frequently reported among the surveyed practitioners. The coexistence of traditional and digital approaches reflects the complex interaction between clinical requirements, practitioner experience, technological accessibility, and economic constraints.

### 4.2. Material Selection and Fabrication Techniques

Material selection remains a fundamental determinant of the clinical performance of provisional restorations. In the present study, composite resin was the most frequently utilized material, followed by acrylic resin. These findings are consistent with previous reports indicating that both materials continue to occupy a central role in provisional prosthodontics because of their favorable balance between cost, availability, handling characteristics, and clinical performance [32,33].

The predominance of composite resin may be explained by its superior esthetic properties, acceptable wear resistance, and ability to facilitate chairside modifications [32,34]. Acrylic resin also maintained a substantial presence within daily practice, likely reflecting its affordability and long-standing clinical familiarity [32,35]. Despite the emergence of digitally manufactured alternatives, conventional materials continue to satisfy the clinical requirements of many provisional restorative situations [23,36].

Interestingly, no significant associations were identified between practice environment and material selection, suggesting that restorative preferences are influenced more strongly by clinical considerations than by geographical factors. Similarly, professional status was not significantly associated with the use of polycarbonate crowns (*p* = 0.051). Given the limited sample size, this result should not be interpreted as evidence of differences among professional categories.

The direct technique represented the most frequently reported fabrication method. This observation is not unexpected, as direct procedures offer immediate restoration delivery, reduced laboratory dependency, and lower treatment costs [34,37]. Nevertheless, the substantial proportion of clinicians utilizing indirect techniques may reflect increasing awareness of the benefits associated with improved polymerization control, enhanced marginal adaptation, and superior esthetic outcomes [36,38].

### 4.3. Digital Transformation and Technology Adoption

One of the most relevant findings of the present study concerns respondents’ generally favorable perceptions of digital technologies within provisional prosthodontics. Most respondents perceived CAD/CAM systems and additive manufacturing technologies as capable of improving restoration quality and expressed openness toward future technological implementation [38]. Nevertheless, only 47.4% of participants provided an affirmative response to the general questionnaire item concerning the current use of digital technologies for provisional restorations. Because the questionnaire did not operationally define this category, the reported proportion may encompass heterogeneous interpretations of digital practice and should not be considered a technology-specific adoption rate.

This finding is consistent with previous international studies demonstrating that positive attitudes toward digital dentistry do not necessarily translate into widespread clinical adoption [27,39]. Van der Zande et al. reported that the implementation of digital technologies among Dutch general dental practitioners was influenced by multiple practitioner-related and practice-related factors, emphasizing the complexity of technology adoption in contemporary dentistry [40]. Similarly, Tran et al. observed that although many dentists in the United Kingdom considered CAD/CAM systems important for the future of dentistry, actual utilization remained limited, with financial investment and uncertainty regarding clinical advantages representing major barriers [41].

More recent evidence suggests a gradual increase in digital integration across different healthcare systems. Krastev et al. reported that 71.3% of Austrian dentists had used CAD/CAM technology at least once and that 51.8% had access to CAD/CAM systems in their workplace. However, cost-related concerns and doubts regarding the superiority of digital workflows over conventional techniques remained important obstacles [23]. Direct comparison with the present findings is limited by differences in the definitions and measurement of digital technology use. Nevertheless, the affirmative response reported by 47.4% of the present participants suggests an ongoing but incomplete integration of digital workflows within the surveyed group.

Comparable trends have been reported in Middle Eastern countries. Al-Ibrahim et al. demonstrated that Saudi dentists generally exhibited positive attitudes toward CAD/CAM technologies and expressed a strong willingness to incorporate digital workflows into clinical practice [42]. Likewise, Radwan et al. found that although practitioners recognized the potential benefits of digital dentistry, implementation was frequently hindered by high initial costs, resistance to change, interoperability issues, and training-related barriers [43]. These observations are consistent with the present findings, in which high equipment and material costs were identified as the principal disadvantage of additive manufacturing technologies and patient affordability was frequently perceived as an important consideration.

The results of the present study also align with those reported by Suganna et al., who observed favorable perceptions of digital dentistry among practitioners but highlighted considerable variability in actual utilization and emphasized the importance of continuing professional education [21]. In the current investigation, scientific congresses, professional meetings, and continuing education courses represented the primary sources of professional information, further supporting the critical role of lifelong learning in facilitating digital transformation.

**Figure 3:**
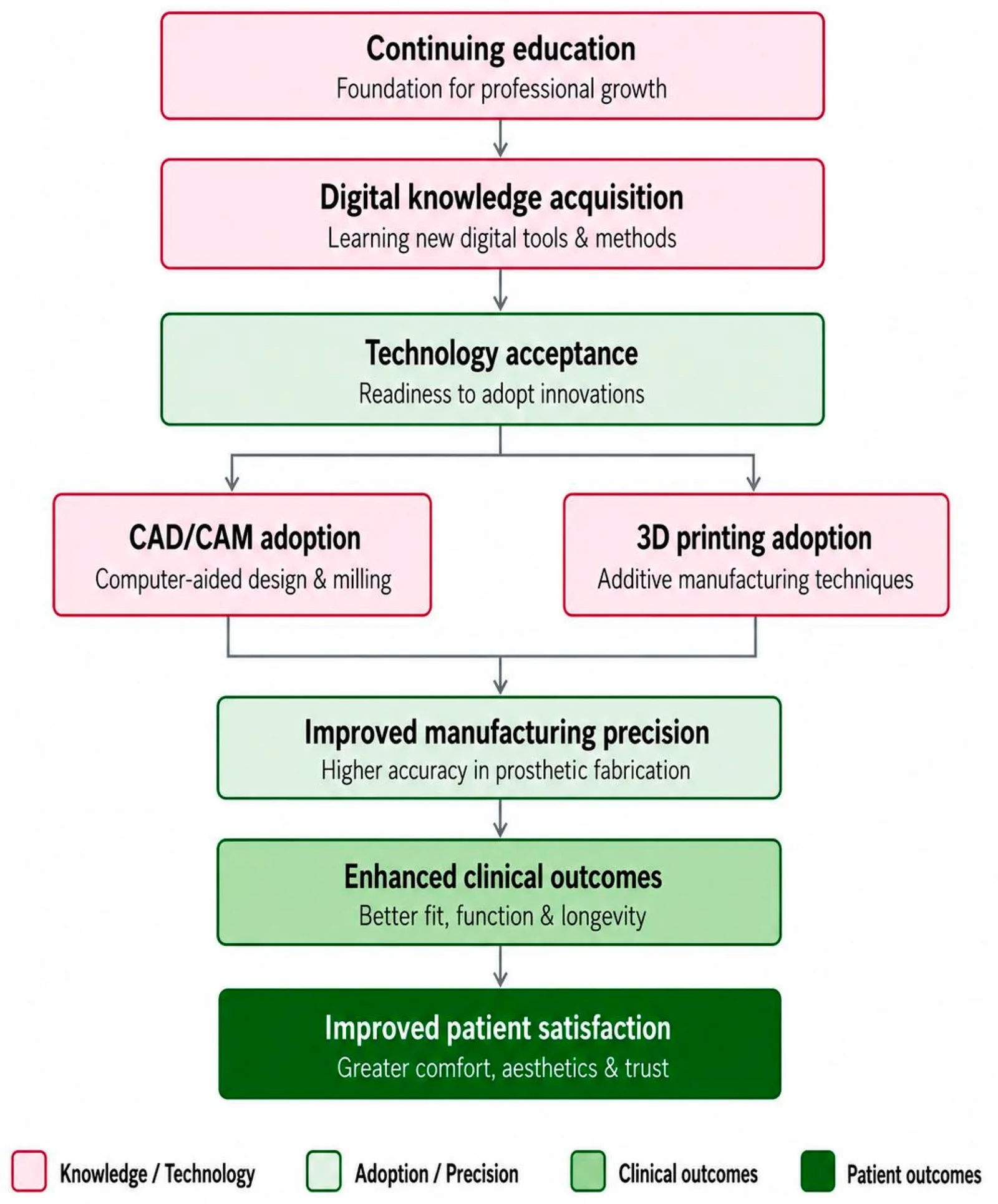
Proposed conceptual framework of factors influencing digital technology adoption in provisional prosthodontics.

Further support for the relationship between professional knowledge and clinical implementation comes from a recent study conducted in China. Zhu et al. showed that among dentists in Jiangsu province, knowledge of digital dental technologies exerted a direct effect on attitude, which in turn directly influenced practice, indicating that knowledge also acted indirectly on clinical implementation through attitude formation [44]. In their sample, CAD/CAM systems were reportedly used by less than half of respondents (42.45%), despite considerably higher levels of theoretical familiarity with the technology, a pattern broadly comparable to the gap between favorable perceptions and actual utilization observed in the present study. These findings support the structural relationship proposed in the conceptual framework presented in Figure 3, wherein continuing education and digital knowledge acquisition precede technology acceptance and subsequent clinical adoption.

A higher degree of digital integration has been reported in Switzerland. Mühlemann et al. found that approximately one-quarter of Swiss dental practices were equipped with chairside CAD/CAM systems and that digital workflows contributed substantially to restorative procedures [45]. Compared with these findings, the surveyed practitioners appear to represent a group undergoing an incomplete transition toward digital workflows.

Recent evidence from Jordan further supports the multifactorial nature of technology adoption. Hatamleh et al. reported that digital dentistry implementation was influenced by access to equipment, availability of materials, and training opportunities, with notable differences observed between dental practitioners and laboratory technicians [24]. These findings reinforce the results of the present study, suggesting that successful implementation of digital prosthodontic workflows depends not only on technological availability but also on educational, organizational, and economic factors.

The potential influence of practice environment on digital technology adoption has also been highlighted in recent evidence from India. Bhatnagar et al. reported measurable disparities in digital adoption between rural and urban dental practitioners in Western India and attributed the gap between theoretical knowledge and clinical implementation to structural barriers, including unequal access to equipment and infrastructure [46]. In the present study, however, practice environment was not significantly associated with digital technology utilization. Given the limited sample size and the exploratory nature of the regression model, the observed estimate for urban practice should not be interpreted as evidence of a meaningful independent association. Larger studies are required to determine whether infrastructural accessibility contributes to urban–rural differences in digital adoption among Romanian dental practitioners.

The exploratory logistic regression analysis did not identify statistically significant independent associations between the investigated practitioner- or practice-related characteristics and digital technology utilization. Given the limited number of outcome events relative to the number of predictor parameters, the individual odds ratios, confidence intervals, and apparent model-performance estimates should not be overinterpreted. The analysis should be regarded solely as hypothesis-generating. Future adequately powered studies should evaluate additional determinants, including institutional support, collaboration with technologically advanced laboratories, availability of digital infrastructure, practice size, volume of prosthodontic activity, access to in-house digital equipment, and financial resources.

Altogether, the descriptive findings suggest that the adoption of digital technologies in provisional prosthodontics may represent a multidimensional process shaped by technological, educational, organizational, and economic considerations. Although favorable attitudes toward innovation were frequently reported, barriers related to cost, training, and infrastructure may limit broader implementation. These observations are descriptive and hypothesis-generating and require confirmation in larger, representative studies using clearly defined technology-specific outcomes.

To facilitate the interpretation of the principal fabrication approaches currently available for provisional restorations, a comparative overview of conventional, CAD/CAM-milled, and 3D-printed workflows is presented in Table 5.

### 4.4. Educational and Economic Considerations Related to Adoption

The descriptive findings highlight continuing professional education and economic feasibility as considerations perceived by respondents to be relevant to digital technology adoption. Scientific congresses and continuing education courses represented the principal sources of professional information among respondents, highlighting the central role of lifelong learning in contemporary restorative practice.

The rapid evolution of restorative materials, digital workflows, and manufacturing technologies requires clinicians to continuously update their knowledge and skills [47,48]. Previous studies have identified insufficient training and limited familiarity with digital technologies as important barriers to implementation [47,49]. Consequently, educational initiatives aimed at strengthening digital competencies may facilitate the responsible integration of emerging technologies into routine clinical practice [48,50].

Economic considerations were also frequently perceived as potential barriers to adoption. Most respondents believed that patient acceptance of digitally fabricated provisional restorations would be influenced by the additional costs associated with these treatments. Similarly, high equipment and material costs were identified as the principal disadvantage of additive manufacturing technologies.

These findings suggest that successful digital transformation depends not only on technological performance but also on affordability for both practitioners and patients [18,51]. Reductions in equipment costs, increased accessibility of digital infrastructure, and broader educational opportunities may therefore accelerate the implementation of digital workflows within restorative dentistry.

### 4.5. Clinical Implications and Conceptual Framework

The descriptive findings are consistent with the hypothesis that digital transformation in provisional prosthodontics represents a multidimensional process involving technological, educational, organizational, economic, and patient-related considerations. Based on the descriptive results of this study and the existing literature, an exploratory conceptual framework was developed to illustrate factors that may influence the integration of digital technologies into provisional restorative practice (Figure 3).

Within this framework, practitioner-related factors include clinical experience, openness toward innovation, and engagement in continuing professional education. Patient-related factors encompass esthetic expectations, perceived treatment value, and financial considerations. Technological factors involve equipment availability, workflow complexity, material performance, and manufacturing capabilities. Finally, educational and organizational factors influence practitioners’ ability to acquire the competencies required for successful implementation.

The complications reported by respondents further highlight the clinical relevance of optimizing provisional restorative workflows. Debonding and fracture were identified as the most frequently encountered complications, emphasizing the continued importance of appropriate material selection, meticulous clinical execution, and evidence-based treatment planning. Additional professional training and collaboration with specialized laboratories were among the most frequently adopted strategies for improving clinical outcomes, reinforcing the importance of continuous professional development and interdisciplinary cooperation.

All these findings suggest that the successful integration of digital technologies requires more than technological acquisition alone. Sustainable implementation depends on coordinated adaptation involving education, infrastructure, economic accessibility, and patient-centered decision-making [52,53]. Such an approach may facilitate the transition toward more predictable, efficient, and individualized provisional prosthodontic treatment [54].

## 5. Limitations

The present study has several limitations. First, the study used non-probability convenience sampling and voluntary participation, which may have introduced selection and non-response bias and limited the generalizability of the findings. Second, the survey assessed self-reported clinical practices and perceptions rather than objectively verified clinical behaviors or patient outcomes. Therefore, the results may be affected by recall, reporting, and social desirability bias and cannot provide evidence regarding the clinical superiority of digital workflows over conventional techniques. In addition, two response options concerning the perceived disadvantages of 3D printing partially overlapped conceptually, as both referred to operator competence. This item-design limitation may have affected respondents’ ability to distinguish between equipment calibration and operator-related requirements and should be considered when interpreting the corresponding response frequencies.

Furthermore, the key outcome variable concerning the current use of digital technologies for provisional restorations was not accompanied by a sufficiently precise operational definition. Respondents may have interpreted this category as including different components, such as CAD/CAM milling, 3D printing, intraoral scanning, or broader digital documentation and communication procedures. This measurement heterogeneity may have resulted in outcome misclassification and cannot be resolved retrospectively. Future questionnaires should evaluate each technology separately and provide an explicit operational definition of a digital fabrication workflow.

Third, the questionnaire did not differentiate complications according to the type of provisional restoration, precluding restoration-specific analyses. Fourth, although the questionnaire included practice environment, professional status, years of clinical experience, and type of practice, it did not collect detailed information regarding practice size, the number of clinicians employed within each practice, the frequency of provisional restoration use, the monthly volume of prosthodontic procedures, the proportion of clinical activity devoted to prosthodontics, or access to in-house digital equipment. These unmeasured practice-level characteristics may have influenced the reported material preferences and digital technology adoption patterns. Future studies should incorporate these variables and use larger samples to permit adequately powered subgroup analyses.

Furthermore, no formal a priori sample-size calculation was performed before questionnaire distribution because the study was designed as an exploratory survey using non-probability convenience sampling. The final sample size was determined by the number of fully completed questionnaires returned by practitioners from the predefined closed distribution list during the study period. Although the response rate was 63.3%, the achieved sample of 95 participants was relatively modest and may not have provided sufficient statistical power to detect small or moderate associations, particularly in subgroup and multivariable analyses. Moreover, because participants were recruited using non-probability convenience sampling, the sample cannot be considered nationally representative. Accordingly, the findings should be interpreted as preliminary and exploratory.

Additionally, the exploratory multivariable logistic regression model included 12 predictor parameters relative to only 45 outcome events, resulting in an events-per-parameter ratio below commonly recommended thresholds. This limited ratio may have reduced the stability of the individual odds ratio estimates and contributed to the wide confidence intervals observed for several predictors. Furthermore, the model underwent no internal or external validation. Accordingly, the odds ratios, confidence intervals, AUC, and Nagelkerke’s R^2^ are sample-specific, unvalidated estimates and should be interpreted solely as exploratory and hypothesis-generating rather than as evidence of stable independent effects or reliable predictive performance.

Finally, the cross-sectional design precludes causal inference regarding factors associated with digital technology utilization.

## 6. Conclusions

Within the limitations of this exploratory cross-sectional survey, composite and acrylic resins were the most frequently reported materials among the participating Romanian dental practitioners, while direct fabrication remained the most commonly used technique. Although respondents generally expressed favorable perceptions of digital technologies, only 47.4% provided an affirmative response to the general item concerning their current use in the fabrication of provisional restorations. This proportion should be interpreted cautiously because digital technology use was not operationally defined in the questionnaire.

The exploratory multivariable analysis did not identify statistically significant independent associations with digital technology utilization. Therefore, the present findings do not establish predictors of digital adoption or demonstrate the clinical superiority of digital workflows over conventional techniques. Instead, they provide preliminary descriptive information regarding current practices and practitioners’ perceptions within the surveyed group. Larger, representative, and adequately powered studies using clearly defined technology-specific outcomes and objective clinical measures are required to evaluate the determinants, implementation, and clinical performance of digital workflows in provisional prosthodontics.

## Figures and Tables

**Table 1:** Distribution of respondents according to practice environment and type of practice.

Practice Environment	Private Practice, *n*	Insurance-Contracted Practice, *n*	Total, *n* (%)
Urban	53	18	71 (74.7)
Rural	13	11	24 (25.3)
Total	66	29	95 (100.0)

**Table 2:** Consolidated distribution of participants’ responses regarding provisional restorative practices and digital technology adoption (*N* = 95).

Survey Item/Domain	Response Option	*n*	%
Materials used for provisional restorations ^1^	Composite resin	74	77.9
	Acrylic resin	62	65.3
	Polycarbonate crowns	51	53.7
	Celluloid crowns	47	49.5
	Metallic provisional restorations	39	41.1
Principal criterion influencing material selection	Cost	28	29.5
	Resistance	26	27.4
	Esthetics	21	22.1
	Fabrication time	20	21.1
Preferred fabrication technique	Direct technique	49	51.6
	Indirect technique	40	42.1
	Direct–indirect technique	6	6.3
Openness toward emerging technologies and materials	Yes, definitely	30	31.6
	Maybe, if proven reliable	24	25.3
	I am not sure	29	30.5
	No, I prefer conventional methods	12	12.6
Principal perceived advantage of 3D-printed provisional restorations	Faster and more efficient fabrication	40	42.1
	Superior adaptation within the oral cavity	39	41.1
	Improved esthetics	11	11.6
	Biocompatibility and safety	5	5.3
Principal perceived disadvantage of 3D printing	High equipment and material costs	56	58.9
	Final quality depends on equipment calibration and operator competence	11	11.6
	Limited material resistance	22	23.2
	Dependence on operator competence	6	6.3
Perceived influence of digital technologies on restoration quality	Significantly	14	14.7
	To a great extent	39	41.1
	Partially	37	38.9
	To a limited extent	5	5.3
Current use of digital technologies for provisional restorations	Yes	45	47.4
	No	50	52.6
Perceived patient acceptance of digitally fabricated provisional restorations	Yes, most patients would	8	8.4
	Yes, but not all patients can afford the additional costs	51	53.7
	No, most patients would prefer conventional provisional restorations for financial reasons	36	37.9
Principal source of professional information	Scientific congresses and professional meetings	48	50.5
	Continuing education courses	19	20.0
	Internet resources	16	16.8
	Scientific journals and textbooks	12	12.6
Principal strategy used to improve clinical outcomes	Additional professional training	42	44.2
	Collaboration with specialized dental laboratories	32	33.7
	Updating equipment and restorative materials	21	22.1
Most frequently encountered complication	Debonding	42	44.2
	Fracture	40	42.1
	Periodontal complications	13	13.7

^1^ Multiple responses were permitted for the item concerning materials used for provisional restorations; therefore, percentages for this item do not sum to 100%. For all other questionnaire items, participants selected a single response option.

**Table 3:** Associations between respondents’ characteristics and the use of provisional restorative materials.

Variable	*p*-Value	Cramer’s V
Practice environment		
Acrylic resin	0.251	0.119
Composite resin	0.864	0.033
Polycarbonate crowns	0.602	0.067
Metallic restorations	0.134	0.154
Celluloid crowns	0.320	0.102
Professional status		
Acrylic resin	0.944	0.040
Composite resin	0.521	0.123
Polycarbonate crowns	0.051	0.286
Metallic restorations	0.846	0.055
Celluloid crowns	0.500	0.158

**Table 4:** Exploratory multivariable logistic regression analysis of factors associated with reported digital technology utilization.

Variable	OR	95% CI	*p*-Value
Male sex	1.74	0.70–4.30	0.231
Urban practice environment	2.21	0.77–6.34	0.139
Private practice	0.38	0.14–1.03	0.056
6–10 years of experience	0.41	0.13–1.35	0.144
>10 years of experience	0.59	0.21–1.65	0.314
Non-use of composite materials	2.75	0.88–8.63	0.084
Non-use of celluloid crowns	0.92	0.36–2.32	0.860
Indirect technique	1.12	0.43–2.93	0.825
Direct–indirect technique	1.38	0.20–9.46	0.744
Uncertainty toward emerging technologies	1.43	0.44–4.63	0.548
Preference for conventional methods	1.26	0.28–5.80	0.763
Conditional openness toward innovation	1.00	0.31–3.28	0.999

Note: OR = odds ratio; CI = confidence interval. Reference categories were female sex, rural practice environment, insurance-contracted practice, 0–5 years of professional experience, use of composite materials, use of celluloid crowns, direct technique, and definite willingness to adopt emerging technologies. The apparent model-performance estimates were AUC = 0.714 and Nagelkerke’s R^2^ = 0.182. Given the limited events-per-parameter ratio and the absence of internal or external validation, all estimates should be interpreted as exploratory and hypothesis-generating.

**Table 5:** Comparative characteristics of conventional, CAD/CAM-milled, and 3D-printed provisional restorations.

Characteristic	Conventional Techniques	CAD/CAM-Milled Restorations	3D-Printed Restorations
Manufacturing process	Direct or indirect manual fabrication	Subtractive manufacturing	Additive manufacturing
Initial investment	Low	High	High
Equipment requirements	Minimal	Milling unit and software	Printer, software, post-processing unit
Production time	Variable	Moderate	Reduced
Marginal adaptation	Operator dependent	High precision	High precision
Esthetic outcomes	Good	Good to excellent	Good to excellent
Mechanical performance	Variable	Excellent	Moderate to good
Material waste	Moderate	High	Minimal
Need for technical expertise	Moderate	High	High
Degree of customization	Moderate	High	High
Accessibility in routine practice	High	Moderate	Moderate
Main limitations	Operator variability	Cost and material waste	Cost and technical demands

## Data Availability

The anonymized data supporting the findings of this study are available from the corresponding authors upon reasonable request.

## Supplementary Materials

The following supporting information can be downloaded at https://www.mdpi.com/article/10.3390/dj14080489/s1, File S1: Questionnaire Assessing Current Clinical Practices and Digital Technology Adoption in Provisional Prosthodontics; Table S1: STROBE Checklist for Reporting Cross-Sectional Studies.

## Abbreviations

The following abbreviations are used in this manuscript:

3D	Three-Dimensional
AUC	Area Under the Receiver Operating Characteristic Curve
CAD/CAM	Computer-Aided Design/Computer-Aided Manufacturing
CI	Confidence Interval
GDP	General Dental Practitioner
OR	Odds Ratio
PEMA	Polyethyl Methacrylate
PMMA	Polymethyl Methacrylate
SPSS	Statistical Package for the Social Sciences
STROBE	Strengthening the Reporting of Observational Studies in Epidemiology
VIF	Variance Inflation Factor

## References

[1] Field J., Wassell R. (2023). Provisional restorations (Part 1). Br. Dent. J..

[2] Field J., Wassell R. (2023). Provisional restorations (Part 2). Br. Dent. J..

[3] Regish K.M., Sharma D., Prithviraj D.R. (2011). Techniques of Fabrication of Provisional Restoration: An Overview. Int. J. Dent..

[4] Patras M., Naka O., Doukoudakis S., Pissiotis A. (2012). Management of Provisional Restorations’ Deficiencies: A Literature Review. J. Esthet. Restor. Dent..

[5] Esquivel J., Gomez Meda R., Villarroel M. (2024). Timing implant provisionalization: Decision-making and systematic workflow. J. Esthet. Restor. Dent..

[6] Monje A., González-Martín O., Ávila-Ortiz G. (2023). Impact of peri-implant soft tissue characteristics on health and esthetics. J. Esthet. Restor. Dent..

[7] Spînu A., Manole F., Burcea A., Albu C.-C., Argint A., Mărcuț L.-F., Brata R.D., Manole A., Bogdan-Andreescu C.F. (2026). Management of Advanced Peri-Implantitis with Staged Explantation and Delayed Re-Implantation in the Esthetic Zone. Dent. J..

[8] Jain S., Sayed M.E., Shetty M., Alqahtani S.M., Al Wadei M.H.D., Gupta S.G., Othman A.A.A., Alshehri A.H., Alqarni H., Mobarki A.H. (2022). Physical and Mechanical Properties of 3D-Printed Provisional Crowns and Fixed Dental Prosthesis Resins Compared to CAD/CAM Milled and Conventional Provisional Resins: A Systematic Review and Meta-Analysis. Polymers.

[9] Sokhal K., Kumar S., Aggarwal R., Kaur I., Thoidingjam B., Deokate A., Nandalur K.R. (2025). Comparative Evaluation of Surface Hardness of Bis-Acryl Composite and Polymethyl Methacrylate-Based Provisional Restorative Materials: An In Vitro Study. Cureus.

[10] Idrissi H.A., Annamma L.M., Sharaf D., Jaghsi A.A., Abutayyem H. (2023). Comparative Evaluation of Flexural Strength of Four Different Types of Provisional Restoration Materials: An In Vitro Pilot Study. Children.

[11] Giti R., Dabiri S., Motamedifar M., Derafshi R. (2021). Surface roughness, plaque accumulation, and cytotoxicity of provisional restorative materials fabricated by different methods. PLoS ONE.

[12] Falahchai M., Rahimabadi S., Khabazkar G., Babaee Hemmati Y., Neshandar Asli H. (2022). Marginal and internal fit and fracture resistance of three-unit provisional restorations fabricated by additive, subtractive, and conventional methods. Clin. Exp. Dent. Res..

[13] Al-Humood H., Alfaraj A., Yang C.C., Levon J., Chu T.G., Lin W.S. (2023). Marginal Fit, Mechanical Properties, and Esthetic Outcomes of CAD/CAM Interim Fixed Dental Prostheses (FDPs): A Systematic Review. Materials.

[14] Titihazan F., Veja I., Zaharia C., Hajaj T., Sinescu C., Constantin G.D., Rominu M. (2025). Comparative Evaluation of Color Stability and Fracture Resistance of CAD/CAM and Chairside Provisional Restorations: An In Vitro Study. J. Funct. Biomater..

[15] Andreescu C.F., Ghergic D.L., Botoacă O., Barbu H.M., Cernusca Mitariu I.S., Pătroi D.N. (2017). The Advantages of High-Density Polymer CAD/CAM Interim Restorations in Oral Implantology. Mater. Plast..

[16] Burcea A., Bănățeanu A.-M., Poalelungi C.V., Forna N., Cumpătă C.N. (2024). Enhanced properties and multifaceted applications of polymethyl methacrylate (PMMA) in modern medicine and dentistry. Rom. J. Oral Rehabil..

[17] Bănățeanu A.M., Cumpătă C.N., Burcea A. (2024). Digital PMMA, When and Which. Rom. J. Oral Rehabil..

[18] Abdulkarim L.I., Alharamlah F.S.S., Abubshait R.M., Alotaibi D.A., Abouonq A.O. (2024). Impact of Digital Workflow Integration on Fixed Prosthodontics: A Review of Advances and Clinical Outcomes. Cureus.

[19] Abduo J., Rasaie V. (2025). Digital Workflows in Prosthodontics. Aust. Dent. J..

[20] Usman M.J., Latha N., Saraswathy A., Makkadan Thachanath H. (2024). Integration of Three-Dimensional Scanning and Computer-Aided Design/Computer-Aided Manufacturing (CAD/CAM) Technology in Routine Prosthodontic Practice: A Cross-Sectional Study in Kerala. Cureus.

[21] Suganna M., Nayakar R.P., Alshaya A.A., Khalil R.O., Alkhunaizi S.T., Kayello K.T., Alnassar L.A. (2024). The Digital Era Heralds a Paradigm Shift in Dentistry: A Cross-Sectional Study. Cureus.

[22] Eggmann F., Blatz M.B. (2024). Recent Advances in Intraoral Scanners. J. Dent. Res..

[23] Krastev T., Payer M., Krastev Z., Cardelles J.F.P., Vegh A., Banyai D., Geczi Z., Vegh D. (2023). The Utilisation of CAD/CAM Technology Amongst Austrian Dentists: A Pilot Study. Int. Dent. J..

[24] Hatamleh M.M., Saleh S.A., Radwan O.I., Shamma H.R.A. (2025). Adoption and challenges of digital dentistry among dentists and dental technicians: A cross-sectional study. J. Prosthodont..

[25] Schnitzler C., Bohnet-Joschko S. (2025). Technology readiness drives digital adoption in dentistry: Insights from a cross-sectional study. Healthcare.

[26] Schnitzler C., Bohnet-Joschko S. (2025). Digital transformation in dentistry: A survey on trends and business implications. Heliyon.

[27] Katyayan M. (2026). Adapting to digital change in Indian dental laboratories. J. Indian Prosthodont. Soc..

[28] Burde A.-V., Baciu S., Manole M. (2019). Assessment of the Use of CAD/CAM Technology Among Romanian Dental Technicians. A Web-Based Survey. Int. J. Med. Dent..

[29] Cozmescu A.F., Cernega A., Mincă D.G., Didilescu A.C., Meleșcanu Imre M., Ripszky Totan A., Pârvu S., Pițuru S.-M. (2025). Embracing Artificial Intelligence in Dental Practice: An Exploratory Study of Romanian Clinicians’ Perspectives and Experiences. Dent. J..

[30] Andronic A., Maniu G., Birlutiu V., Popa M. (2025). Utilization Patterns and Implementation Barriers in Adoption of Teledentistry Within Romanian Dental Practice. Healthcare.

[31] Law No. 190/2018; Lege Privind Măsuri de Punere în Aplicare a Regulamentului (UE) 2016/679 al Parlamentului European și al Consiliului din 27 Aprilie 2016 Privind Protecția Persoanelor Fizice în Ceea ce Privește Prelucrarea Datelor cu Caracter Personal și Privind Libera Circulație a Acestor Date. Monitorul Oficial al României, Partea I, nr. 651: Bucharest, Romania, 2018. https://www.dataprotection.ro/servlet/ViewDocument?id=1685.

[32] Astudillo-Rubio D., Delgado-Gaete A., Bellot-Arcís C., Montiel-Company J.M., Pascual-Moscardó A., Almerich-Silla J.M. (2018). Mechanical Properties of Provisional Dental Materials: A Systematic Review and Meta-Analysis. PLoS ONE.

[33] Barqawi L., Kanot S. (2024). A comparative clinical performance of polymethyl methacrylate (PMMA) and urethane dimethacrylate (UDMA) materials in provisional fixed prostheses. Cureus.

[34] Alfallaj H., Jamjoom F.Z., Alzaid A.A., Alqarni H. (2023). Polytetrafluoroethylene tape spacer for direct splinted teeth-supported provisional restoration: A dental technique. Heliyon.

[35] Singh P., Shenoy A., Nallaswamy D., Maiti S. (2024). Comparative evaluation of microbial adhesion on provisional crowns fabricated with milled polymethyl methacrylate (PMMA) and conventional acrylic resin: A prospective clinical trial. Cureus.

[36] Yılmaz G., Yeşil Z. (2025). A new era in provisional restorations: Evaluating marginal accuracy and fracture strength in additive, subtractive and conventional techniques. BMC Oral Health.

[37] Hasanzade M., Zabandan D., Mosaddad S.A., Habibzadeh S. (2023). Comparison of marginal and internal adaptation of provisional polymethyl methacrylate restorations fabricated by two three-dimensional printers: An in vitro study. Dent. Res. J..

[38] Elsareef S.S., Azer A.S., Morsy N. (2024). Evaluation of fracture resistance and marginal fit of implant-supported interim crowns fabricated by conventional, additive and subtractive methods. BMC Oral Health.

[39] Nayakar R., Sardesai P., Killedar S., Patil A., Kakodker M. (2022). Knowledge, awareness and practices of the use of digital technology in dentistry among postgraduate students and dental practitioners in India: A cross-sectional study. J. Clin. Diagn. Res..

[40] van der Zande M.M., Gorter R.C., Aartman I.H., Wismeijer D. (2015). Adoption and use of digital technologies among general dental practitioners in the Netherlands. PLoS ONE.

[41] Tran D., Nesbit M., Petridis H. (2016). Survey of UK dentists regarding the use of CAD/CAM technology. Br. Dent. J..

[42] Al-Ibrahim I.K., Alshammari F.A., Alanazi S.M., Madfa A.A. (2023). The Attitude of Saudi Dentists Towards CAD/CAM in Restorative Dentistry. Open Dent. J..

[43] Radwan H.A., Alsharif A.T., Alsharif M.T., Aloufi M.R., Alshammari B.S. (2023). Digital technologies in dentistry in Saudi Arabia: Perceptions, practices and challenges. Digit. Health.

[44] Zhu F., Yu H., Wang Z., Lu X., Meng X., Nie R. (2025). Knowledge, Attitude, and Practice Regarding Digital Dental Technologies Among Dentists in Jiangsu Province. Healthcare.

[45] Mühlemann S., Sandrini G., Ioannidis A., Jung R.E., Hämmerle C.H.F. (2019). The use of digital technologies in dental practices in Switzerland: A cross-sectional survey. Swiss Dent. J..

[46] Bhatnagar S., Pai Khot A.J., Dhokar A., Juvekar T.C., Desai V.B. (2026). Knowledge, Attitude, and Practice of Digital Dentistry Among Dentists in Rural and Urban Clinical Settings in Western India: A Descriptive Cross-Sectional Study. Front. Oral Health.

[47] Choukse V., Kunturkar A., Aidasani A.N., Gottlieb A.M., Agrawal R., Bumb P.P. (2023). Survey of Indian dental professionals regarding the use of computer-aided design/computer-aided manufacture (CAD/CAM) technology. Cureus.

[48] Ohyama H., Duong M.L., Yancoskie A.E., Smiley A.B., Syed A.Z., Reddy M.S., Bencharit S. (2025). Challenges and opportunities in implementing digital technology in dental curriculum: A review and perspective. Cureus.

[49] Fernandes G.V.O., Nassani L.M. (2023). The implementation of CAD/CAM technology at schools of dentistry: A short communication. Enviro Dent. J..

[50] Ishida Y., Kuwajima Y., Kobayashi T., Yonezawa Y., Asack D., Nagai M., Kondo H., Ishikawa-Nagai S., Da Silva J., Lee S.J. (2022). Current implementation of digital dentistry for removable prosthodontics in US dental schools. Int. J. Dent..

[51] Gawali N., Shah P.P., Gowdar I.M., Bhavsar K.A., Giri D., Laddha R. (2024). The evolution of digital dentistry: A comprehensive review. J. Pharm. Bioallied Sci..

[52] Sorrentino R., Zarone F., Cantile T., Mastrosimone A., Cervino G., Ruggiero G. (2024). The Use of Digital Tools in an Interdisciplinary Approach to Comprehensive Prosthodontic Treatments. Prosthesis.

[53] Toser I., Faur A.B., Anghel-Lorinți A.E., Jivănescu A. (2025). Case report: A comprehensive digital workflow to enhance predictability and precision with fixed dental prostheses in the posterior region. Front. Dent. Med..

[54] Joda T., Balmer M., Jung R.E., Ioannidis A. (2024). Clinical use of digital applications for diagnostic and treatment planning in prosthodontics: A scoping review. Clin. Oral Implants Res..

