# Supplementary material for: Current Clinical Practices and Digital Technology Adoption in Provisional Prosthodontics: A Cross-Sectional Survey Among Romanian Dental Practitioners
Source: Dent J (Basel). 2026 Aug 6;14(8):489. doi: 10.3390/dj14080489 (PMC13511265; doi:10.3390/dj14080489)
Supplement: Supplementary file 1 [file dentistry-14-00489-s001.zip › File S1.pdf]

Supplementary Material S1

File S1. Questionnaire Assessing Current Clinical Practices and Digital Technology Adoption in Provisional Prosthodontics

Information for Participants

Dear Colleague,

You are invited to participate in an anonymous survey investigating current clinical practices and the adoption of digital technologies in provisional prosthodontics among Romanian dental practitioners.

Participation in this study is entirely voluntary. The questionnaire is anonymous, and no personally identifiable information will be collected, stored, or processed. The information provided will be analyzed only in aggregate form and used exclusively for scientific research purposes and publication.

Completion of the questionnaire requires approximately **5–10 minutes**. Participants may discontinue the survey at any time before submitting their responses.

By completing and submitting this questionnaire, you confirm that you have read the information provided above and voluntarily consent to participate in this study.

Questionnaire

1. Sex

- □ Male
- □ Female

2. Practice environment

- □ Rural
- □ Urban

3. Type of practice

- □ Private practice
- □ Insurance-contracted practice

4. Years of clinical experience

- □ 0–5 years
- □ 6–10 years
- □ More than 10 years

5. Professional status

- □ Resident

- □ General Dental Practitioner (GDP)
- □ Specialist Dentist

If you are a Specialist Dentist, please indicate your specialty:

- □ Prosthodontics
- □ Endodontics
- □ Periodontology
- □ Pediatric Dentistry
- □ General Dentistry
- □ Other specialty (Oral Surgery, Orthodontics and Dentofacial Orthopedics, Oral and Maxillofacial Surgery, etc.)

6. Which materials do you routinely use for provisional restorations? ***(Multiple answers may be selected.)***

- □ Acrylic resin
- □ Composite resin
- □ Polycarbonate crowns
- □ Metallic provisional restorations
- □ Celluloid crowns

7. What is the primary criterion influencing your choice of material for provisional restorations?

- □ Resistance
- □ Esthetics
- □ Cost
- □ Fabrication time

8. Which fabrication technique do you routinely use for provisional restorations?

- □ Direct technique
- □ Indirect technique
- □ Direct–indirect technique

9. Would you be willing to adopt emerging technologies or materials for provisional restorations in the future?

- □ Yes, definitely
- □ Maybe, if proven reliable
- □ I am not sure
- □ No, I prefer conventional methods

10. In your opinion, what are the main advantages of 3D-printed provisional restorations?

- □ Superior adaptation within the oral cavity
- □ Improved esthetics
- □ Faster and more efficient fabrication
- □ Biocompatibility and safety

11. In your opinion, what are the main disadvantages of 3D-printed provisional restorations?

- □ High equipment and material costs
- □ Final quality depends on equipment calibration and operator competence
- □ Limited material resistance
- □ Dependence on operator competence

12. To what extent do you believe that digital technologies influence the quality of provisional restorations (e.g., adaptation, esthetics, and durability)?

- □ Significantly
- □ To a great extent
- □ Partially
- □ To a limited extent

13. Do you currently use digital technologies for the fabrication of provisional restorations?

- □ Yes
- □ No

14. Do you believe that patients would be willing to receive digitally fabricated provisional restorations despite the additional costs involved?

- □ Yes, most patients would
- □ Yes, but not all patients can afford the additional costs
- □ No, most patients would prefer conventional provisional restorations for financial reasons

15. What are your primary sources of professional information?

(Select one answer.)

- □ Scientific congresses and professional meetings
- □ Continuing education courses
- □ Scientific journals and textbooks
- □ Internet resources

16. Which measure have you recently implemented to improve provisional prosthodontic treatment?

(Select one answer.)

- □ Additional professional training
- □ Updating equipment and restorative materials
- □ Collaboration with specialized dental laboratories

17. What is the most common complication associated with provisional restorations in your clinical practice?

- □ Fracture
- □ Debonding
- □ Periodontal complications
