# Supplementary material for: Current Clinical Practices and Digital Technology Adoption in Provisional Prosthodontics: A Cross-Sectional Survey Among Romanian Dental Practitioners
Source: Dent J (Basel). 2026 Aug 6;14(8):489. doi: 10.3390/dj14080489 (PMC13511265; doi:10.3390/dj14080489)
Supplement: Supplementary file 1 [file dentistry-14-00489-s001.zip › Table_S1_STROBE.pdf]

dentistry journal

MDPI

Table S1. STROBE Checklist for Reporting Cross-Sectional Studies

Manuscript: Current Clinical Practices and Digital Technology Adoption in Provisional Prosthodontics: A Cross-Sectional Survey among Romanian Dental Practitioners

Item No.	Recommendation	Reported on Section
**Title and Abstract**		
1 (a)	Indicate the study's design with a commonly used term in the title or the abstract.	*Title; Abstract ("cross-sectional survey")*
1 (b)	Provide in the abstract an informative and balanced summary of what was done and what was found.	*Abstract*
**Introduction**		
2	Explain the scientific background and rationale for the investigation being reported.	*Section 1, Introduction, ¶ 1–5*
3	State specific objectives, including any prespecified hypotheses.	*Section 1, Introduction, final ¶*
**Methods**		
4	Present key elements of study design early in the paper.	*Section 2.1, Study Design*
5	Describe the setting, locations, and relevant dates, including periods of recruitment, exposure, follow-up, and data collection.	*Section 2.1 (January–March 2025); Section 2.2*
6 (a)	Give the eligibility criteria, and the sources and methods of selection of participants.	*Section 2.2, Study Population and Sampling*
7	Clearly define all outcomes, exposures, predictors, potential confounders, and effect modifiers. Give diagnostic criteria, if applicable.	*Section 2.4, Questionnaire Development and Validation*
8	For each variable of interest, give sources of data and details of methods of assessment.	*Section 2.4; self-reported structured questionnaire*
9	Describe any efforts to address potential sources of bias.	*Section 2.4 (expert panel review, pilot testing); Section 5, Limitations*
10	Explain how the study size was arrived at.	*Section 2.2 (150 questionnaires distributed); Section 5, Limitations (no a priori sample size calculation)*
11	Explain how quantitative variables were handled in the analyses. If applicable, describe which groupings were chosen and why.	*Section 2.5, Statistical Analysis*

12 (a)	Describe all statistical methods, including those used to control for confounding.	*Section 2.5, Statistical Analysis*
12 (b)	Describe any methods used to examine subgroups and interactions.	*Section 2.5 (multivariable logistic regression)*
12 (c)	Explain how missing data were addressed.	*Section 2.2 (incomplete questionnaires excluded)*
12 (d)	If applicable, describe analytical methods taking account of sampling strategy.	*Convenience sampling; addressed in Section 5, Limitations*
12 (e)	Describe any sensitivity analyses.	*Section 2.5 (variance inflation factor assessment)*
**Results**		
13 (a)	Report numbers of individuals at each stage of the study.	*Section 3.1 (150 distributed; 95 completed; 63.3% response rate)*
13 (b)	Give reasons for non-participation at each stage.	*Section 2.2 (incomplete questionnaires excluded)*
14 (a)	Give characteristics of study participants and information on exposures and potential confounders.	*Sections 3.1–3.2; Figures 1–2; Table 1*
14 (b)	Indicate the number of participants with missing data for each variable of interest.	*Section 2.2 (no missing data among the 95 included responses)*
14 (c)	Summarize follow-up time.	*Not applicable (cross-sectional design)*
15	Report numbers of outcome events or summary measures.	*Sections 3.3–3.13; Tables 2–15*
16 (a)	Give unadjusted estimates and, if applicable, confounder-adjusted estimates and their precision.	*Section 3.9; Table 11 (adjusted ORs with 95% CIs)*
16 (b)	Report category boundaries when continuous variables were categorized.	*Table 11, table note (reference categories specified)*
16 (c)	If relevant, translate estimates of relative risk into absolute risk for a meaningful time period.	*Not applicable*
17	Report other analyses done—e.g., subgroup, interaction, and sensitivity analyses.	*Sections 3.4–3.8 (χ^2/Cramer's V); Section 3.9 (AUC, Nagelkerke's R^2)*
**Discussion**		
18	Summarize key results with reference to study objectives.	*Section 4.1, Principal Findings*
19	Discuss limitations of the study, taking into account sources of potential bias or imprecision. Discuss both direction and magnitude of any potential bias.	*Section 5, Limitations*
20	Give a cautious overall interpretation considering objectives, limitations, multiplicity of analyses, results from similar studies, and other relevant evidence.	*Sections 4.1–4.5*
21	Discuss the generalizability (external validity) of the study results.	*Section 5, Limitations*

Other Information		
22	Give the source of funding and the role of the funders for the present study.	*Funding statement*
